# Evaluating and Classifying Gentleness in VR-Based Surgical Simulation: A VR + fNIRS Study

**DOI:** 10.3390/s26082388

**Published:** 2026-04-13

**Authors:** Suveyda Sanli, Hasan Onur Keles

**Affiliations:** 1 Department of Biomedical Engineering, Ankara University, Ankara 06560, Turkey; ssanli@ankara.edu.tr; 2 Brain Research Center (AUBAUM), Ankara University, Ankara 06900, Turkey; 3 Neuroscience and Neurotechnology Center of Excellence Brain Research Center (NOROM), Ankara University, Ankara 06560, Turkey

**Keywords:** fNIRS, virtual reality, skill assessment, gentleness

## Abstract

Gentleness, defined as the ability to handle tissues delicately while minimizing unnecessary force, is a critical indicator of surgical proficiency. Objective and real-time assessment of gentleness in virtual reality (VR)-based training can improve the understanding of both psychomotor and cognitive components of surgical skill. This study evaluates and classifies participants’ gentleness during VR-based laparoscopic simulations using fNIRS-derived hemodynamic features. Twenty-three volunteers with no prior laparoscopic experience performed a VR-based double-grasper task while hemodynamic activity over frontal and motor cortical regions was recorded using eighteen fNIRS channels. In parallel, subjective workload (NASA-TLX), error counts, and gentleness performance score (GPS) were collected. Temporal features, including slope, root mean square, and standard deviation, were extracted from the fNIRS signals and used to train multiple machine learning classifiers. Performance labels were binarized into low and high groups using median splits of the gentleness performance score. Models were evaluated using stratified 5-fold cross-validation. Results revealed stronger right-frontal HbO activity and increased left-motor HbR responses in the low-performance group, suggesting higher cognitive effort and less efficient motor strategies. Across classifiers, slope-based features consistently outperformed variability- and amplitude-based metrics. The highest classification performance was achieved using HbR slope features with Random Forest classifiers (accuracy ≈ 0.85, AUC up to 0.93). These findings highlight the potential of fNIRS-based metrics for automated performance assessment in VR surgical training.

## 1. Introduction

Virtual reality (VR) simulators have become increasingly integrated into medical education [1]. Over the past decade, their use has expanded in the field of surgical training, especially, and VR platforms continue to maintain strong popularity as training tools [2,3]. These platforms are now routinely implemented for basic psychomotor skill acquisition, procedural rehearsal, team-based simulation, and competency-based assessment. However, their overall effectiveness and the mechanisms through which they enhance surgical performance remain subjects of ongoing discussion [4,5,6]. Specifically, the underlying neural and cognitive mechanisms remain to be fully understood [7]. Combining the VR headsets with neuroimaging techniques offers an important approach to uncovering the neural and cognitive mechanisms underlying surgical skill acquisition and performance during VR-based training [8,9,10].

Surgical training has traditionally measured success with skills like speed, accuracy, and error rates. However, an equally vital skill, namely gentleness, is often overlooked in these assessments [11]. This study focuses specifically on this critical skill. While traditional metrics such as task completion time focus on efficiency, gentleness is a distinct qualitative dimension representing the economy of force and tissue preservation. A surgeon may be fast (efficient) but traumatic (not gentle). Gentleness, therefore, is not merely a byproduct of speed but a high-order cognitive motor skill requiring precise force feedback integration and inhibitory control of motor output. VR simulators present a valuable tool for assessing essential surgical competencies like gentleness, which is a fundamental indicator of competence across all surgical domains, as excessive force can cause tissue damage and prolong patient recovery. Importantly, surgical gentleness encompasses more than motor execution alone; it involves sustained attention, anticipatory planning, inhibition of unnecessary force, and precise regulation of movement under limited sensory feedback [12,13,14]. Because of its multidimensional nature, numerous surgical education frameworks have emphasized the need to objectively quantify gentleness and incorporate it into modern assessment tools. Gentleness has been incorporated into procedural assessment frameworks, particularly in minimally invasive surgery, where excessive force may lead to unintended tissue damage, postoperative complications, and prolonged recovery times. Evidence shows that surgical skill is a strong predictor of patient outcomes, and gentleness is one of the primary contributors to overall surgical competency [15].

The Fundamentals of Laparoscopic Training (FLS) curriculum is a mandatory component of laparoscopic education and must be completed prior to board certification in some countries [16]. Safe surgery has been defined by principles such as gentle handling of tissues, meticulous hemostasis, avoidance of dead space, and adherence to precise surgical technique. Among these principles, gentleness has increasingly gained recognition as a measurable and essential aspect of operative performance assessment.

Despite its importance, surgical skill evaluation traditionally relies on subjective assessments within an apprenticeship-model training paradigm [16,17]. Such evaluations lack objective structure and fail to provide meaningful, real-time feedback to trainees [18]. In contrast, proficiency-based simulation training has demonstrated superior outcomes, leading to fewer errors and complications in the operating room and improving trainee performance across multiple metrics. As a result, many surgical training programs have adopted simulation-based curricula to support skill acquisition and assessment [19]. Given the growing emphasis on competency-based education and the clear clinical relevance of gentleness, there is a need for objective and scalable tools that quantify this skill [11]. Addressing this gap is the importance of the present study. There is a growing need to utilize VR simulators in combination with functional neuroimaging modalities to investigate the neural mechanisms underlying motor learning, cognitive workload, and decision-making during simulated surgery.

In minimally invasive surgery, appropriate manipulation forces, bimanual coordination, and gentleness toward soft tissue are essential to avoid unintended tissue damage and to accomplish stable and precise handling [17,20,21,22]. Traditional metrics such as error counts or task duration cannot fully capture these subtle aspects of motor behavior. Therefore, assessing gentleness, defined as the ability to perform precise manipulation with minimal unnecessary force, has become a parameter in objective skill quantification. The double-grasper manipulation task, which requires users to transfer a soft, deformable object (balloon) using a pair of grasper tools, provides a natural environment for quantifying gentleness. Successful performance demands bilateral coordination, adequate pressure control, and strategic motor planning to avoid tearing, dropping, or excessively deforming the object. Consequently, the gentleness performance score (GPS) obtained from this task is considered a sensitive indicator of soft-tissue handling skill, and can differentiate novice behavior from more controlled manipulation strategies [11,23].

Functional near-infrared spectroscopy (fNIRS) provides a portable and ecologically valid functional neuroimaging method for investigating cortical hemodynamics in naturalistic task environments. Recent studies employing fNIRS for surgical skill assessment can be broadly categorized into three themes. First, a significant body of work has focused on cognitive workload, where prefrontal cortex (PFC) activation is consistently higher in novices and decreases with skill acquisition, reflecting more efficient executive control in experts [16,24,25,26]. Second, several studies have investigated motor cortex activation patterns, linking them to task complexity, bimanual coordination, and the development of motor automaticity [26,27,28]. Third, a growing but still limited number of studies have begun to apply machine learning (ML) techniques to fNIRS data to classify skill levels (e.g., novice vs. expert) or predict performance scores [29,30,31]. However, these studies have used data from traditional simulators or non-VR environments. There are also some studies combining VR and the surgical skill assessment without neuroimaging [32,33,34]. Most critically, there is a distinct lack of research applying ML algorithms to fNIRS data within immersive VR-based surgical training environments, particularly for predicting specific skill dimensions, like gentleness, from cortical activation patterns. By integrating VR performance metrics with fNIRS-derived cortical activation patterns, it becomes possible not only to assess how well participants perform but also to characterize how they allocate cognitive and motor resources to achieve gentle manipulation. Therefore, the main research objective of this study is to investigate whether gentleness during VR-based laparoscopic manipulation can be objectively quantified from cortical hemodynamic activity measured by fNIRS, with the broader aim of establishing a quantitative and standardized evaluation framework to support competency-based training and future professional certification in VR-based surgical education.

In this paper, we investigate the cerebral hemodynamic prediction of gentleness by dividing gentleness performance scores into two groups (high vs. low). Classification is performed using both prefrontal and motor cortical activation features, demonstrating that optical signals contain reliable information to discriminate levels of gentleness during VR-based surgical training. In addition, we examine the relationship between subjective workload and gentleness, showing that higher gentleness is associated with reduced perceived workload.

The proposed methodology highlights that combining fNIRS imaging with machine learning provides an objective and skill-relevant metric that complements subjective assessments in VR environments. This neuroimaging-based framework offers quantitative and standardized measures that can support professional certification and surgical education during VR training. This is the first study conducted with the participants, and future work will expand the study to include expert and novice surgeons using the insights gained from this initial phase.

## 2. Materials and Methods

### 2.1. Subjects

The study involved 23 volunteer participants with no prior laparoscopic experience. The participants had an average age of 27 ± 6 years. All participants were right-handed. All participants provided written informed consent prior to the experimental session, in accordance with the Declaration of Helsinki. The study protocol was reviewed and approved by the Ankara University Human Research Ethics Committee (Approval No: 2024000333-1), and data were collected between 20 June 2024 and 20 August 2025.

### 2.2. Experimental Protocol

**Figure 1:**
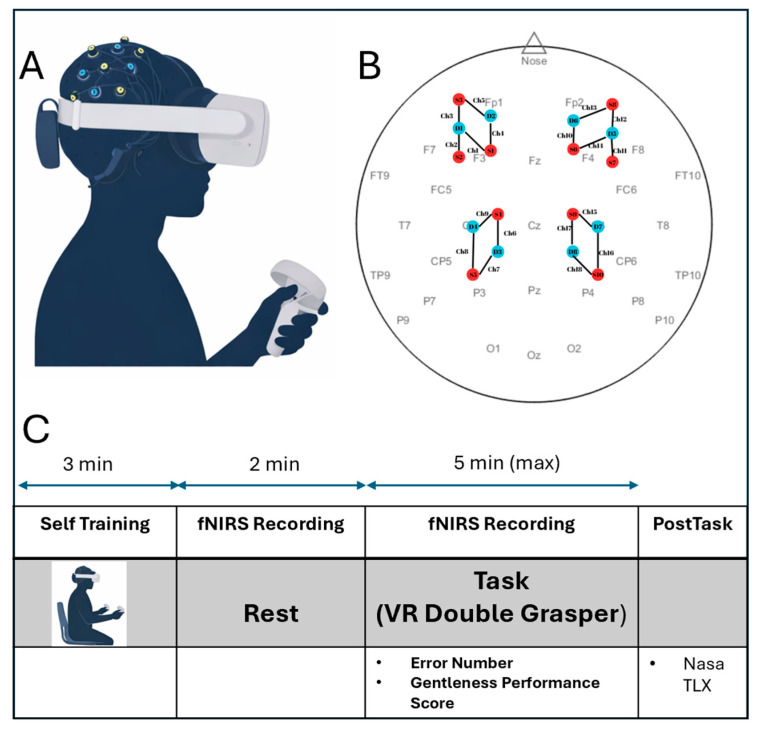
Experimental setup and fNIRS sensor configuration during VR-based laparoscopic training. (**A**) A participant wearing the fNIRS cap and VR headset while performing the laparoscopic simulation task using a handheld controller. (**B**) Optode layout showing the placement of sources and detectors over frontal and motor cortices following the international EEG 10–20 system. (**C**) Timeline of the experiment illustrating the calibration period, VR task execution, and recording duration during free laparoscopic training.

Participants performed the Laparoscopic Double Grasper Task in a VR environment while fNIRS data were recorded in a single session. Prior to data collection, a brief 3-minute practice trial was conducted to familiarize participants with the virtual setting. To evaluate surgical gentleness and precision, a 3D virtual reality simulator (the Laparoscopic Double Grasper Task) was developed in Unity 3D and deployed on the Meta Quest 3 headset. The simulator was based on a previously developed screen-based laparoscopic simulation platform and was adapted and extended to an immersive VR environment to support the gentleness assessment paradigm used in this study [11]. The wireless setup allowed natural interaction and real-time tracking of instrument movements, while continuous visual feedback enhanced spatial awareness and depth perception throughout the task. Participants first completed a 2-min resting-state baseline while keeping their eyes open and minimizing movement, which served as a reference for task-related activity (Figure 1). They then performed the 5-min Laparoscopic Double Grasper Task, manipulating a soft, tissue-like balloon with two virtual graspers in the VR environment.

Laparoscopic Double Grasper Task: The Laparoscopic Double Grasper Task was designed to evaluate participants’ bimanual coordination and gentle tissue handling in a simulated laparoscopic environment. The task required participants to use two laparoscopic graspers to transfer a soft, balloon-shaped object representing fragile biological tissue between two target boxes within a virtual operating room. The balloon stiffness was set to 0.1 based on prior VR surgical simulation studies to approximate soft-tissue behavior and to allow discrimination between gentle and forceful manipulation through slippage and rupture events. In the virtual operating room, two boxes were placed on a table and separated by a central frame. A soft, balloon-shaped object representing biological tissue was initially positioned in the left box.

**Figure 2:**
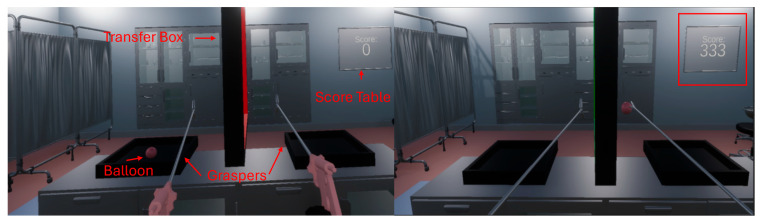
VR double-grasp task used in laparoscopic simulation. The VR environment requires users to grasp and transfer virtual objects (ball) between two target areas using two laparoscopic controllers. Performance is scored automatically in real time based on task completion and errors (e.g., drops or missed grasps), and the cumulative score is displayed on the screen.

Participants grasped the balloon with one grasper, transferred it through the inside of the central transfer box, and placed it into the opposite box using the other grasper. One complete transfer cycle consisted of moving the balloon from one box to the other and back again. Excessive grasping force caused the balloon to burst, producing an audible feedback signal and being recorded as an error. If the grasping force was insufficient, the balloon slipped away, causing time loss within the five-minute task period. Performance during the task was quantified using a single metric referred to as the gentleness performance score (GPS). The GPS was calculated automatically in real time by the simulator and reflected both task success (completed transfer) and movement precision. Specifically, successful transfers performed into the center of the transfer box were awarded up to 50 points; accurate placements into the left or right box yielded an additional 25 points. A real-time score display in the VR scene provided performance feedback. The visual components of the VR environment are shown in Figure 2. 

### 2.3. Data Collection

We used a wearable continuous-wave fNIRS device (Brite, Artinis Medical Systems, Elst, The Netherlands). The device is a continuous-wave system using two wavelengths (760 nm and 850 nm) to record back-reflected light intensity. The fNIRS system comprised 10 sources and 8 detectors arranged in 2 × 4 and 2 × 5 probe layouts over the frontal and motor cortical regions, respectively. The resulting 18 channels were symmetrically divided, with 9 channels placed over each hemisphere. Each hemisphere was monitored by 5 prefrontal (frontal) channels and 4 motor cortex channels (Figure 1). Optode–scalp contact and signal quality were rigorously verified prior to beginning the recording session. Sources and detectors are separated by approximately 30 mm (i.e., long-separation channels). During the fNIRS recording, the number of errors was also recorded. All behavioral and fNIRS signals were synchronized to ensure precise temporal alignment. After the task, participants completed the NASA-TLX survey to rate perceived workload across mental, physical, temporal demand, performance, effort, and frustration, each on a 20-point visual analog scale.

### 2.4. Data Analysis

Homer3 v1.87.0 and MATLAB R2017b (The MathWorks, Inc., Natick, MA, USA) were used for the preprocessing and analysis of the fNIRS data. Channels with poor signal quality were identified based on raw light intensity amplitude thresholds outside the acceptable range (1.7772 × 10^−4^ to 1.9 arbitrary units). Following channel pruning, the signal was converted into optical density (OD). After a visual inspection of the data, the wavelet-based method was applied to correct for motion artifacts [35]. To remove high-frequency oscillations and physiological noise, the corrected OD signal was processed using a sixth-order Butterworth low-pass filter with a frequency range of 0.01–0.5 Hz. Finally, the changes in optical density were converted into concentration changes in oxygenated hemoglobin (HbO) and deoxygenated hemoglobin (HbR) by employing the Modified Beer–Lambert Law [36].

To assess localized cortical activity, we calculated the standard deviation of oxyhemoglobin changes within non-overlapping 10-s windows for each measurement channel. Since a stronger evoked hemodynamic response typically increases the signal variability within a given window, we used these standard deviation values as an indicator of prefrontal activation. This method is preferable to using the window mean in certain cases, such as when the evoked response is short-lived and followed by a subsequent drop in the signal [16,37].

We also applied this windowed standard deviation to identify significant motion artifacts, specifically those with amplitudes exceeding those caused by normal physiological activity. A smaller standard deviation would indicate a less severe artifact. For each channel, we computed the standard deviation across all 10-s windows and then determined the median absolute deviation (MAD) of those values. Any window whose standard deviation fell more than 4.5 MAD units above the median was labeled as an outlier and removed from further analysis.

From the preprocessed HbO and HbR signals, several statistical features were computed for the 5-min task period, including the standard deviation, root mean square (RMS), and slope values. Feature extraction was performed within 10-s windows, and each window was treated as an independent data sample. To investigate whether fNIRS-derived features could discriminate between different gentleness, performance scores were divided into low and high groups and used as binary class labels. All features were normalized using StandardScaler, and model evaluation employed Group 5-fold cross-validation with a 20% test split in order to prevent the same subject’s data from occurring in both the training and test sets.

**Figure 3:**
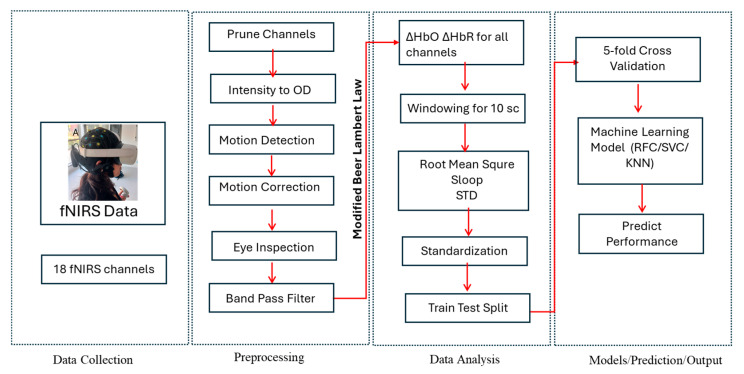
The workflow for fNIRS data acquisition, preprocessing, and machine learning classification.

Several machine learning classifiers were trained and compared, including Random Forest, Support Vector Machine (SVM) and K-Nearest Neighbors (KNN). GridSearchCV was used for hyperparameter optimization. Model performance was evaluated using accuracy, F1-score, precision, and area under the ROC curve (AUC), and results were visualized using confusion matrices. Figure 3 summarizes the experimental workflow from raw fNIRS data acquisition to performance prediction.

#### 2.4.1. NASA-TLX and Gentleness Performance Score Evaluation

To explore the relationship between subjective workload and objective task performance, Pearson correlation analyses were conducted between each NASA-TLX subscale and gentleness performance score. Subscales included mental demand, physical demand, temporal demand, performance, effort, and frustration, as well as the total workload score. Correlation strength and direction were quantified using r values, with statistical significance set at *p* < 0.05.

#### 2.4.2. Statistical Analysis

When conducting a regression analysis comparing 2 numerical variables, linear fit with analysis of variance was used. For the descriptive results comparing two groups, such as completion time v game experience, completion time v laparoscopy experience, NASA total v game experience, and NASA total v laparoscopy experience, HbO changes contained non-paired data. In order to assess the statistical significance of the difference between two groups of non-paired results, we used the non-parametric Kolmogorov test.We did not utilize null hypotheses whose rejection would have required corrections for multiple comparisons or false discovery.

## 3. Results

**Figure 4:**
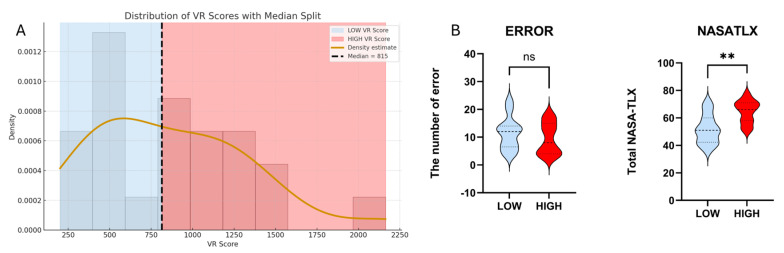
(**A**) Histogram of total gentleness performance scores (GPS) (n = 23) showing a wide variability. Participants were divided into LOW- and HIGH-GPS groups based on the median value (median = 815). (**B**) Comparison of task performance metrics between LOW and HIGH groups. The number of errors did not differ significantly between groups (ns), whereas the HIGH-GPS group reported significantly higher overall workload on the NASA-TLX Statistical significance is indicated as follows: ns = not significant; ** *p* < 0.01.

We report behavioral, subjective, and neurophysiological results from a VR-based laparoscopic training task involving 23 participants. The results demonstrate the relationship between gentleness performance scores and perceived workload as assessed by the NASA-TLX and its subscales. Hemodynamic differences between high- and low-gentleness groups are examined across prefrontal and motor cortical regions. In addition, machine learning classification of gentleness is summarized using fNIRS-derived cortical features, specifically SLOPE, RMS, and STD. The predictive performance of the best-performing fNIRS-based models is further illustrated through confusion matrices, highlighting the effectiveness of HbR slope-based features classified using Random Forest and K-Nearest Neighbors approaches. A total of 23 participants were included in the VR performance analysis. Figure 4 shows that the distribution of total gentleness performance scores exhibited wide variability, ranging from 201 to 2163. Based on the median value (Median = 815), participants were divided into two performance groups: LOW VR (≤815; n = 12) and HIGH VR (>815; n = 11). This median-based split was used for subsequent group-level comparisons.

Figure 4B shows that the HIGH-GPS group did not exhibit a significant reduction in the number of errors compared to the LOW-GPS group (ns; *p* > 0.05), indicating that higher VR performance was not necessarily associated with fewer mistakes. However, the HIGH-GPS group reported significantly higher NASA-TLX workload scores (*p* < 0.01), suggesting that participants who performed better experienced greater perceived cognitive and physical demand during the task.

**Figure 5:**
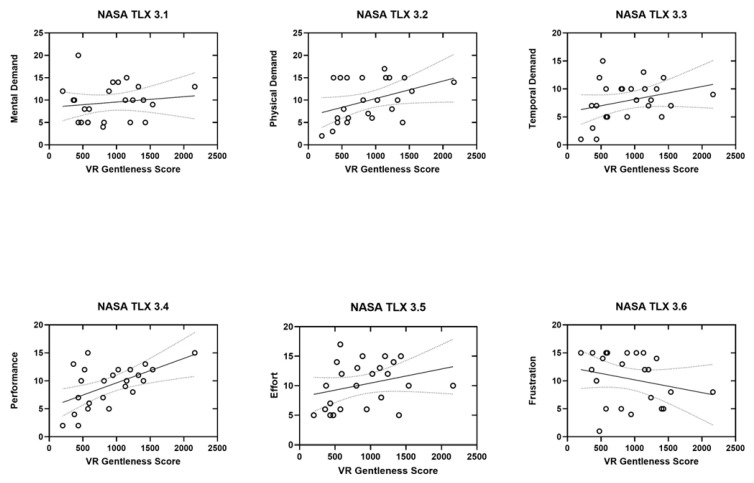
Relationship between gentleness performance score and NASA-TLX subscales. Scatter plots with linear regression show associations between gentleness performance score and perceived workload. Gentleness performance score was positively correlated with Mental Demand (3.1), Physical Demand (3.2), Temporal Demand (3.3), Performance (3.4), and Effort (3.5), whereas Frustration (3.6) showed a slight negative trend. Shaded areas indicate the 95% confidence interval of the regression.

Figure 5 shows that regression analyses between gentleness performance score and NASA-TLX subscales showed significant relationships for Physical Demand (*p* = 0.05) and Performance (*p* = 0.005). Trends in the same direction were observed for Mental Demand, Temporal Demand, and Effort; however, these did not reach significance. Frustration showed a weak negative relationship with the gentleness performance score. The analysis of the total NASA-TLX score also revealed a significant association with the gentleness performance score (*p* = 0.01).

**Figure 6:**
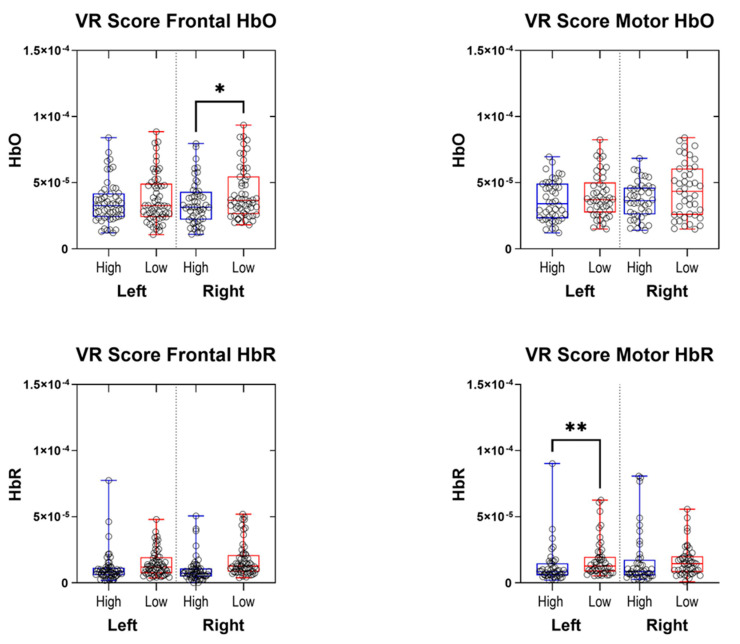
Group-level comparison of frontal and motor cortex hemodynamic responses between high and LOW-GPS groups. Box plots show oxygenated (HbO) and deoxygenated (HbR) hemoglobin concentrations for left and right frontal and motor regions. Participants were divided into HIGH- vs. LOW-GPS groups based on the median gentleness performance score. Statistical significance is indicated as follows: * *p* < 0.05; ** *p* < 0.01.

For hemodynamic data analysis, we used the concentration change of HbO and HbR for 18 fNIRS channels, with 5 prefrontal (frontal) channels and 4 motor cortex channels in each hemisphere. fNIRS data, which was previously preprocessed by segmenting the continuous signal into non-overlapping 10-second windows and subjected to rigorous artifact rejection (excluding any window whose standard deviation exceeded 4.5 Median Absolute Deviation (MAD) values away from the median), revealed significant and lateralized modulations in cerebral hemodynamic responses. Each data point (circle) in Figure 6 represents the aggregated result of a single channel. Specifically, analysis of HbO revealed that in the Right Frontal Cortex, the HIGH-GPS group exhibited a significantly lower concentration of HbO compared to the LOW-GPS group (*p* < 0.05). Conversely, no significant differences were observed for HbO in the Left Frontal Cortex or either motor cortex. A distinct finding emerged in the HbR concentrations: in the Left Motor Cortex, the HIGH-GPS group showed a highly significant increase in HbR concentration compared to the LOW-GPS (*p* < 0.01). No other significant differences were found for HbR in the right motor or bilateral frontal cortices.

Table 1 shows that classification performance of fNIRS features in the gentleness performance score was evaluated using three classifiers (RFC, SVC, and KNN) across different feature types (SLOPE, STD, and RMS) and HbO/HbR signals.

For the HbO signal, the highest classification performance under subject-level cross-validation was obtained using slope-based features with the Random Forest classifier (RFC). This configuration achieved an accuracy of 0.7346, an F1-score of 0.6957, a precision of 0.7633, a recall of 0.6391, and an AUC of 0.8153. The k-Nearest Neighbors (KNN) model performed slightly better in accuracy (0.7500) and recall (0.8254) but had a lower AUC (0.7987). The Support Vector Classifier (SVC) yielded the weakest performance among slope-based models (accuracy = 0.6770, AUC = 0.7368). Models trained with STD and RMS features of HbO showed lower classification accuracy, ranging from 0.63 to 0.71, with AUC scores predominantly below 0.75. Among these, HbO STD + KNN (accuracy = 0.7093, AUC = 0.7466) and HbO RMS + RFC (accuracy = 0.6433, AUC = 0.7079) were the best within their respective feature categories.

The HbR Slope + RFC configuration produced the best overall performance among all tested models, reaching an accuracy of 0.8567, an F1-score of 0.8365, a precision of 0.9126, a recall of 0.7722, and a high AUC of 0.9349. In contrast, the KNN classifier on HbR slope features achieved significantly lower accuracy (0.7472) and AUC (0.8106). Within the STD and RMS feature sets, performance was weaker, with accuracy values between 0.62 and 0.73. The HbR STD + KNN model was the best among these (accuracy = 0.7289, AUC = 0.7877). Crucially, HbR slope features consistently and substantially outperformed all other HbR feature types as well as the best HbO-based models.

**Figure 7:**
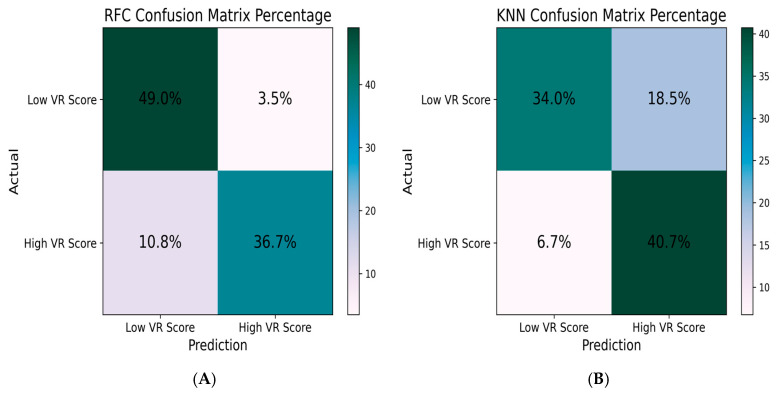
Confusion matrices of the best-performing fNIRS-based models for gentleness classification. (**A**) HbR slope-based features classified using RFC. (**B**) HbR slope-based features classified using KNN.

Based on the normalized confusion matrix results (Figure 7), slope-based models demonstrated the highest overall classification performance across the evaluated machine learning algorithms. The SVC–Slope configuration showed the most balanced classification outcome, with correct prediction rates of 38.5% for the LOW-GPS group and 47.6% for the HIGH-GPS group. RMS- and STD-based feature models resulted in comparatively lower correct classification rates across classifiers, with higher levels of misclassification observed particularly in the LOW-GPS category. Across all confusion matrices, HIGH-GPS predictions were consistently classified with greater accuracy than LOW-GPS.

## 4. Discussion

The main scientific insight of this study is that gentleness, a clinically critical yet traditionally under-quantified skill, exhibits a measurable cortical hemodynamic signature that can be captured using fNIRS and decoded using machine learning during VR-based surgical training. Our findings provide evidence that gentleness can be assessed using a combination of VR performance metrics and fNIRS-derived cortical signals. While previous VR-based laparoscopic training studies have primarily relied on behavioral and task-based outcomes alone, the present study extends this work by incorporating neural measures of cognitive effort and motor control. To our knowledge, this is the first study to classify gentleness in a simulated laparoscopic task using machine learning applied to hemodynamic features, and to directly compare feature types and model performance within the same framework. These results offer new insight into how cortical activity patterns relate to subtle variations in motor strategy during VR-based surgical training.

The significant relationships observed between the gentleness performance score and NASA-TLX subscales indicate a close link between objective motor control and subjective workload perception during VR-based surgical simulation. Specifically, higher gentleness performance scores were associated with lower physical strain and improved perceived performance, suggesting that participants who applied smoother and more controlled tool forces experienced reduced physical demand and greater task efficiency. The significant association with the total NASA-TLX score further supports the notion that refined psychomotor control contributes to a lower overall workload. These findings align with previous studies demonstrating that experts exert lower and more consistent tool–tissue interaction forces, reflecting both improved motor coordination and cognitive regulation during task execution. The integration of the gentleness metric with subjective workload measures thus provides complementary insights into the interplay between cognitive effort and motor precision, reinforcing the potential of force-derived indices as valid indicators of surgical expertise and training progress in immersive VR environments.

The hemodynamic patterns observed in this study provide important insight into the neural processes underlying gentle tissue manipulation during VR-based laparoscopic simulation. The increased HbO and HbR responses observed in the low-performance group, particularly within the right frontal cortex and left motor cortex, suggest a reliance on greater cognitive control and motor correction mechanisms during task execution. This aligns with established evidence showing that early skill acquisition is associated with heightened activation in frontal executive regions as learners engage working memory, error monitoring, response inhibition, and attentional control to maintain task demands. In contrast, participants classified with HIGH-GPS demonstrated reduced cortical activation in both frontal and motor regions, suggesting more efficient neural resource allocation and greater procedural fluency. Although both groups had no prior experience to the laparoscopy or VR gaming, the HIGH-GPS group exhibited neural patterns and classification results more typical of advanced learners. This discrepancy may be explained by inter-individual differences in baseline psychomotor aptitude [38] or prior experience with video gaming [25]. It is documented that individuals with extensive video gaming experience demonstrate superior visuospatial mapping and faster transition to ‘procedural automaticity’ [39,40,41,42,43]. Consequently, the high-gentleness group might have utilized pre-existing neural pathways for spatial navigation and fine motor control, allowing for more efficient neural resource allocation from the outset [44,45].

The significant lateralization effects observed in the left motor cortex further support the interpretation that task proficiency was associated with more automatic motor control. The left hemisphere is known to be dominant in bimanual coordination and sensorimotor integration, particularly in tasks requiring precision and stability, and reduced activation in this region has previously been linked with expert-level performance [30,46]. Similar reductions in prefrontal engagement have been reported in experienced surgeons and skilled tool users, reflecting a progression from deliberate control toward procedural automation as task demands become internalized [47,48]. The current findings, therefore, extend this neuromotor efficiency framework to VR-based assessment of gentleness, demonstrating that subtle behavioral differences in force modulation are mirrored by measurable changes in cortical activation.

Together, these results suggest that fNIRS-derived hemodynamic signals may serve as meaningful biomarkers of surgical gentleness, capturing differences in motor strategy that may not be evident from behavioral metrics alone. The observed relationship between reduced prefrontal engagement and gentler manipulation supports the potential use of neuroadaptive feedback systems to guide learners toward more efficient and safer instrument handling patterns during simulation-based training.

The fNIRS-based classification of gentleness performance, evaluated under a subject-level cross-validation scheme, confirmed that slope features derived from both HbO and HbR signals provided the most discriminative power. Crucially, deoxyhemoglobin (HbR) dynamics emerged as the most robust biomarker. The HbR Slope + Random Forest (RFC) model achieved the highest generalizable performance with an accuracy of 0.8567 and a strong AUC of 0.9349. In contrast, the best HbO-based model (HbO Slope + KNN) reached a lower accuracy of 0.7500 and AUC of 0.7987. Notably, when evaluated on unseen subjects, HbR slope features substantially outperformed their HbO counterparts, and both slope-based feature sets consistently surpassed static or variability-based features (STD, RMS). These findings underscore that the temporal evolution of hemodynamic response, particularly the decrease in HbR concentration indicative of increased neural activity and vasodilation, is a more informative and subject-invariant signal for differentiating levels of fine motor control and movement smoothness during VR-based surgical tasks.

The superior performance of HbR features compared to HbO aligns with evidence that deoxygenated hemoglobin changes more directly reflect localized neuronal activation and are less affected by systemic physiological artifacts [49]. The slope parameter captures the rate of task-related cortical activation, offering a sensitive index of how effectively participants regulate force and precision while interacting with the virtual environment [50].

From a methodological perspective, the superior performance of KNN may be attributed to its non-parametric nature and ability to model non-linear relationships between hemodynamic patterns and subjective workload levels. While more complex algorithms (e.g., RFC, SVC) performed comparably on certain feature sets, KNN consistently provided the most balanced precision–recall profile, suggesting robustness for small-sample datasets.

From a methodological perspective, the choice of validation strategy critically influenced model ranking and performance interpretation. Under the window-based evaluation (Supplementary Table S1), KNN often showed competitive, high performance, likely leveraging local temporal similarities within subjects. However, under the subject-level validation, Random Forest (RFC) demonstrated superior robustness and generalizability, particularly for the most informative HbR slope features. This suggests that RFC’s ensemble learning and inherent feature selection were better at capturing the underlying subject-invariant physiological patterns, reducing overfit to individual-specific noise. While KNN’s non-parametric nature is advantageous for complex relationships, its performance in our setting appeared more susceptible to inflation from subject-dependent temporal correlations. Therefore, for future fNIRS-based prediction tasks where generalizability to new individuals is paramount, ensemble methods like Random Forest evaluated with subject-level protocols are recommended.

The comparison between validation methods highlights that subject-level cross-validation is crucial for realistic performance estimates. While window-based evaluation inflated metrics (e.g., HbR Slope + RFC AUC: 0.9656), subject-level validation provided conservative but reliable scores (AUC: 0.9349). Notably, KNN’s performance dropped substantially under subject-level testing, revealing sensitivity to subject-specific temporal dependencies. This confirms that subject-level protocols are necessary to identify truly generalizable models, and in our case, underscore HbR slope features paired with Random Forest as the most robust combination.

Integrating these findings with behavioral metrics, such as gentleness performance score, underscores the complementary nature of neurophysiological and psychomotor indicators in assessing cognitive–motor efficiency. Participants demonstrating smoother and more controlled tool-force modulation also exhibited lower subjective workload and stronger HbR slope responses, supporting the notion that optimized motor control is accompanied by efficient cortical resource allocation. Together, these outcomes highlight the potential of multimodal frameworks, combining VR performance data and fNIRS features for objective and adaptive assessment of surgical training performance.

Overall, these results suggest that HbR slope dynamics serve as a robust neurophysiological marker of fine motor performance, while HbO slope responses provide complementary information about overall cortical oxygenation during task execution. Together, they support the utility of fNIRS in capturing performance-related cortical changes associated with gentleness performance score.

This study has several limitations that should be considered when interpreting the findings. First, the number of participants was relatively small, which limits the statistical power of the analyses and may have constrained the performance and generalizability of the machine learning models. A larger sample size would allow more robust model training and validation, increase confidence in classification outcomes, and potentially reveal subtler neurobehavioral differences related to skill level. Furthermore, a comprehensive assessment of baseline motor aptitude or visuomotor proficiency was not conducted. The haptic feedback provided by the VR simulator was limited and did not fully replicate the tactile characteristics of real laparoscopic tissue interaction, which may have influenced gentleness-related behavioral and neural responses. The median split was used as a practical step for initial binary classification, and we acknowledge that this approach simplifies the continuous nature of gentleness performance. Future work will aim to directly predict continuous performance scores using regression-based models. The absence of short-separation channels represents a methodological limitation, as systemic physiological artifacts could not be explicitly regressed due to hardware constraints. Additionally, we did not quantify confounding factors such as prior gaming or fine motor experience, which may influence novice performance. Future studies will include expert surgeons and broader demographics to enhance generalizability.

## 5. Conclusions

This study demonstrates that gentleness during VR-based laparoscopic simulation can be quantified using fNIRS-derived hemodynamic features and machine learning classification. Task-related hemodynamic activity in frontal and motor regions differed between participants with high- and low-gentleness performance scores, indicating that fNIRS measures are sensitive to performance-related neural differences during laparoscopic tasks performed in a virtual reality environment. The successful classification of gentleness using slope-based features highlights the potential of incorporating temporal neural metrics into automated performance assessment frameworks. As VR training platforms continue to evolve, the integration of real-time neurophysiological monitoring may support the development of adaptive feedback systems capable of promoting more efficient motor strategies and safer surgical behaviors. Future work with larger and more diverse samples, including experienced participants (e.g., surgeons), baseline motor aptitude testing, higher-fidelity haptics, and longitudinal study designs, will be essential for refining these methods and evaluating their applicability in real-world surgical education.

## Figures and Tables

**Table 1:** Performance Metrics of Machine Learning Models Classified by fNIRS Features.

Features (VR)	Classifier	Accuracy	F1 Score	Precision	Recall	AUC Score
hbo slope	RFC	0.7346	0.6957	0.7633	0.6391	0.8153
hbo slope	SVC	0.6770	0.6628	0.6570	0.6686	0.7368
hbo slope	KNN	0.7500	0.7582	0.7010	0.8254	0.7987
hbr slope	RFC	0.8567	0.8365	0.9126	0.7722	0.9349
hbr slope	SVC	0.6348	0.6409	0.6010	0.6864	0.7007
hbr slope	KNN	0.7472	0.7632	0.6872	0.8580	0.8106
hbo std	RFC	0.6699	0.6445	0.6594	0.6302	0.7109
hbo std	SVC	0.6728	0.6647	0.6471	0.6834	0.7374
hbo std	KNN	0.7093	0.7113	0.6728	0.7544	0.7466
hbr std	RFC	0.6812	0.6333	0.6975	0.5799	0.7441
hbr std	SVC	0.6208	0.6390	0.5829	0.7071	0.6585
hbr std	KNN	0.7289	0.7089	0.7231	0.6953	0.7877
hbo rms	RFC	0.6433	0.6265	0.6228	0.6302	0.7079
hbo rms	SVC	0.6334	0.6287	0.6055	0.6538	0.7120
hbo rms	KNN	0.6362	0.6457	0.6005	0.6982	0.6879
hbr rms	RFC	0.6517	0.6013	0.6585	0.5533	0.6736
hbr rms	SVC	0.6643	0.6667	0.6306	0.7071	0.7170
hbr rms	KNN	0.6643	0.6490	0.6443	0.6538	0.7239

## Data Availability

The data presented in this study are available on request from the corresponding author.

## Supplementary Materials

The following supporting information can be downloaded at: https://www.mdpi.com/article/10.3390/s26082388/s1, Table S1. Classification performance using a window-based evaluation.
