# Supplementary material for: Evaluating and Classifying Gentleness in VR-Based Surgical Simulation: A VR + fNIRS Study
Source: Sensors (Basel). 2026 Apr 13;26(8):2388. doi: 10.3390/s26082388 (PMC13119916; doi:10.3390/s26082388)
Supplement: Supplementary file 1 [file sensors-26-02388-s001.zip › sensors-4118691-supplementary.pdf]

Supp. Table S1. Classification performance using a window-based evaluation.

Features	Classifier	Accuracy	F1 Score	Precision	Recall	AUC Score
HBO SLOPE	RFC	0.7692	0.7724	0.8000	0.7467	0.8834
	SVC	0.8601	0.8718	0.8395	0.9067	0.9071
	KNN	0.8392	0.8535	0.8171	0.8933	0.9134
HBR SLOPE	RFC	0.8881	0.8904	0.9155	0.8667	0.9656
	SVC	0.8252	0.8428	0.7976	0.8933	0.9157
	KNN	0.8881	0.8961	0.8734	0.9200	0.9325
HBO STD	RFC	0.7343	0.7397	0.7606	0.7200	0.8084
	SVC	0.7133	0.7285	0.7237	0.7333	0.7888
	KNN	0.7692	0.7975	0.7386	0.8667	0.8508
HBR STD	RFC	0.6713	0.6466	0.7414	0.5733	0.7969
	SVC	0.6573	0.6667	0.6806	0.6533	0.7871
	KNN	0.8462	0.8514	0.8630	0.8400	0.9196
HBO RMS	RFC	0.7622	0.7671	0.7887	0.7467	0.8173
	SVC	0.8042	0.8228	0.7831	0.8667	0.8780
	KNN	0.8042	0.8182	0.7975	0.8400	0.8698
HBR RMS	RFC	0.6783	0.6515	0.7544	0.5733	0.7971
	SVC	0.7343	0.7500	0.7403	0.7600	0.8257
	KNN	0.7902	0.7917	0.8261	0.7600	0.8790
